# Characterization of *Pipistrellus pygmaeus* Bat Virome from Sweden

**DOI:** 10.3390/v14081654

**Published:** 2022-07-28

**Authors:** Harindranath Cholleti, Johnny de Jong, Anne-Lie Blomström, Mikael Berg

**Affiliations:** 1 Section of Virology, Department of Biomedical Sciences and Veterinary Public Health, Swedish University of Agricultural Sciences (SLU), P.O. Box 7028, 750 07 Uppsala, Sweden; anne-lie.blomstrom@slu.se (A.-L.B.); mikael.berg@slu.se (M.B.); 2 Swedish Biodiversity Centre (CBM), Department of Urban and Rural Development, Swedish University of Agricultural Sciences (SLU), P.O. Box 7016, 750 07 Uppsala, Sweden; johnny.de.jong@slu.se

**Keywords:** bat, metagenomics, virome, coronavirus, Sweden

## Abstract

Increasing amounts of data indicate that bats harbor a higher viral diversity relative to other mammalian orders, and they have been recognized as potential reservoirs for pathogenic viruses, such as the Hendra, Nipah, Marburg, and SARS-CoV viruses. Here, we present the first viral metagenomic analysis of *Pipistrellus pygmaeus* from Uppsala, Sweden. Total RNA was extracted from the saliva and feces of individual bats and analyzed using Illumina sequencing. The results identified sequences related to 51 different viral families, including vertebrate, invertebrate, and plant viruses. These viral families include *Coronaviridae, Picornaviridae, Dicistroviridae, Astroviridae, Hepeviridae, Reoviridae, Botourmiaviridae, Lispviridae, Totiviridae, Botoumiaviridae, Parvoviridae, Retroviridae, Adenoviridae*, and *Partitiviridae,* as well as different unclassified viruses. We further characterized three near full-length genome sequences of bat coronaviruses. A phylogenetic analysis showed that these belonged to alphacoronaviruses with the closest similarity (78–99% at the protein level) to Danish and Finnish bat coronaviruses detected in *Pipistrellus* and *Myotis* bats. In addition, the full-length and the near full-length genomes of picornavirus were characterized. These showed the closest similarity (88–94% at the protein level) to bat picornaviruses identified in Chinese bats. Altogether, the results of this study show that Swedish *Pipistrellus* bats harbor a great diversity of viruses, some of which are closely related to mammalian viruses. This study expands our knowledge on the bat population virome and improves our understanding of the evolution and transmission of viruses among bats and to other species.

## 1. Introduction

Bats belong to the order Chiroptera and represent the second most diverse mammals after rodents, comprising over 1423 recognized species [1,2]. They are widely distributed land animals and are spread across the world, with the exception of Antarctica. Bats feed on a varied diet that includes nectar, fruit, pollen, insects, fish, and blood. They can navigate and hunt in the dark due to their capabilities of echolocation and flight [3], and they have been identified to play key roles in ecosystems through pollination, seed dispersal, and the protection of economically important crops, as well as producing guano that is used as fertilizer [4,5].

Bats have, however, also been shown to be natural reservoirs of a variety of viruses, and viral spill-over events from bats to humans and/or animals have led to infectious disease outbreaks [6,7], including outbreaks of severe acute respiratory syndrome coronavirus (SARS-CoV), Middle East respiratory coronavirus (MERS-CoV), SARS-CoV-2, Nipah virus, Ebola, and henipaviruses [8,9,10,11,12,13]. Most viral infections in bats do not cause any symptoms in the bats themselves, probably, due to their tight control of immune responses and high basal expression of defense genes such as interferons and interferon-stimulated genes [14,15]. The unique ecological, behavioral, feeding, and genetic or immunological characteristics may favor bats as natural reservoirs of a large variety of viruses [6]. Moreover, several factors including body condition, sex, social and reproductive factors, and the habitat of bats are believed to influence viral transmission [3,16]. There is an increasing number of bats in urban areas due to anthropogenic land changes, and this leads to closer contact between bats and livestock and humans which amplifies the risk of zoonotic spillovers [17]. This underlines the importance of characterizing bat virome to be able to identify viruses with cross-species potential [6,7,11,18,19,20,21].

Viral metagenomics is a target-free and unbiased approach that has been proven to be a powerful tool for characterizing viral nucleic acids from any type of sample and has also enabled the identification of unknown viruses [22,23]. Several bat metagenomic projects have reported a large number of bat-borne viruses, including some highly divergent viruses in different bat species from different locations including European countries, for example, Denmark [24], France [25], Finland [26], Germany [27], Croatia [28], and Switzerland [29]. These studies have also reported viruses that can potentially infect mammalian species. Despite the limited sampling, the viral composition is highly variable among geographic locations and bat species. This further highlights the importance of understanding the evolution and spread of emerging bat-borne viruses. Approximately 41 bat species have been identified in Europe [30], and 19 species have been reported in Sweden [31]. Recently, Lwande et al., 2022, sequenced a partial genome of alphacoronavirus from *Myotis daubentonii* bats from southern Sweden [32]. However, apart from this, little data are available about viruses circulating in the Swedish bat population. Therefore, in this study, we used viral metagenomics to identify viruses in fecal and saliva samples collected from *Pipistrellus pygmaeus* (Leach, 1825) bats from two locations in Uppsala, Sweden. In addition, through these studies, near full-length genomes of bat corona- and picornaviruses were characterized.

## 2. Materials and Methods

### 2.1. Sample Collection

In total, 10 Soprano pipistrelle, *Pipistrellus pygmaeus*, were trapped at two different locations at Hammarskog, a nature reserve in Uppsala, Sweden (geographic coordinates: location 1: - 59.7760, 17.5853 and location 2: - 59.7798, 17.5629). The bats were trapped using mist nets when leaving their roosts. The field work was performed during June and July 2020. Four of the bats were collected at location 1 and six at location 2. The species were determined by a bat specialist, and the saliva samples were collected from individuals using swabs that were then put in PBS solution. Prior to sampling, the bats were placed in a cloth bag to keep warm, and any droppings (fecal samples) were collected from the cloth bag. Through this approach, feces were obtained from all bats at location 1 and from 5 of the 6 bats trapped at location 2. The bats were released immediately after the sample collection. The swabs and feces were stored on ice in the field, and then transferred to −80 °C until further use.

### 2.2. Nucleic Acid Extraction and Sequencing

The fecal samples were transferred to Precellys CK14 tubes containing 1.4 mm ceramic beads (Bertin Corp., Rockville, MD, USA) and 450 μL of TRIzol™ LS reagent (Invitrogen, Carlsbad, CA, USA). Then, they were mechanically homogenized using a Precellys Evolution tissue homogenizer (Bertin Corp., Rockville, MD, USA), according to the following protocol: 4 cycles with 30 s/cycle and 30 s pause between each cycle at 6500 RPM. This step was performed under cold conditions using liquid nitrogen. The supernatant was collected after the centrifugation at 13,000× *g* for 5 min at 4 °C and filtered using ultracentrifuge membrane filters (0.45 μM; Utrafree®-MC centrifugal Filter, Darmstadt, Germany) at 13,000× *g* for 4 min. The total RNA was extracted using a combination of TRIzol and GenJet RNA extraction columns (Thermo Fisher Scientific, Waltham, MA, USA) and eluted in 40 μL of nuclease-free water. The quality and quantity of the RNA was measured using a 4200 TapeStation system (Agilent Technologies, Santa Clara, CA, USA) and a Qubit high sensitivity RNA assay kit (Invitrogen, Carlsbad, CA, USA). The extracted RNA was stored at −80 °C until further use. For the saliva samples, 200 μL of the PBS/swab solution was used, and RNA was extracted using a combination of TRIzol and GeneJet RNA extraction kit as previously described. Next, 5–10 μL of RNA from each bat (No. 10) was pooled based on sample type and geographical location, and a total of 6 pools (up to 3 RNA samples/pool) were obtained. The pooled RNA was treated with DNase using an RNAse-free DNase set (Qiagen, Venlo, The Netherlands) and purified with an RNeasy MinElute Cleanup kit (Qiagen, Hilden, Germany). Ribosomal RNA was depleted using a RiboMinus Eukaryotic System v2 (Thermo Fisher Scientific, Waltham, MA, USA), according to the manufacturer’s instructions. The RNA clean-up was performed with an RNeasy MinElute Cleanup kit (Qiagen, Hilden, Germany) as before and eluted in nuclease-free water in the final step. Complementary DNA synthesis and SPIA amplification of RNA were performed using an Ovation RNA-seqV2 system (Tecan, Männedorf, Switzerland), as per the manufacturer’s protocol, and the products were purified using a GeneJet PCR Purification Kit (Thermo Fisher Scientific, Waltham, MA, USA) and eluted in 30 μL of nuclease-free water. The quality and size distribution of the products were estimated using a 4200 TapeStation System (Agilent Technologies, Santa Clara, CA, USA). The amplified ovation products (6 amplicons) were pooled again to 3 samples based on sample type and location as before and submitted to the SNP&SEQ Technology platform in Uppsala, Sweden for library preparation and sequencing. The libraries were prepared using a SMARTer ThruPLEX DNA-seq library preparation kit (Takara Bio USA, Inc. San Jose, CA, USA), and high-throughput sequencing (HTS) was performed with 150 cycles of paired-end sequencing on one SP flow cell using a NovaSeq 6000 system and v1 sequencing chemistry (Illumina Inc., San Diego, CA, USA).

### 2.3. Bioinformatic Analysis

The raw data from the NovaSeq 6000 sequencing run were quality checked with fastp (0.23.2) by trimming adapter sequences, low sequencing quality tails, and removing duplicate reads [33]. Then, the filtered data were processed in two ways: First, the paired-end reads were classified using the Kaiju web server [34], a fast and sensitive classifier for metagenomic data, using a BLAST-nr database (nr+euk, 2021-03) with an e-value of 0.001, and the viral read counts assigned to each viral family were reported. Second, the filtered data were assembled by a *de novo* assembler, Megahit (v.1.2.9) [35], and then the generated contigs were taxonomically classified by BLASTx using Diamond (2.0.14) [36], and aligned against the BLAST-nr database. The Diamond output files were imported to MEGAN (6.18.0) [37] for visualization. The longer viral contigs were extracted and further analyzed to achieve near full-length or partial genomic sequences. The filtered reads were mapped against full-length genomes or partial genomes using Bowtie2 (2.3.5.1) [38] to estimate the base coverage, and consensus sequences were extracted. The nucleotide positions for 5′ and 3′ termini and open reading frames (ORFs) were annotated using the closest reference sequence from the NCBI GenBank and the NCBI ORF finder tool.

### 2.4. PCR and Sanger Sequencing of Viral Genomes

Viral contigs were used to manually design specific primers to confirm the presence of a virus as well as to amplify the missing regions of the selected viruses from the unprocessed total RNA. Reverse transcription was performed using a SuperScript III reverse transcriptase (Invitrogen, Carlsbad, CA, USA), and PCR amplification was performed using AmpliTag Gold^TM^ DNA Polymerase (Applied Biosystems, Foster City, CA, USA). Thermal cycling was initiated with a denaturation step at 95 °C for 10 min, followed by 35 cycles of 95 °C for 30 s, 59–60 °C for 30 s, 72 °C for 1 min, and a final extension at 72 °C for 7 min. The PCR primer pairs used in this study, for coronavirus (BtCoV/F-MV2/P.pyg/SE/2020), were 5′-TGAAGGCTGAAGGTGATGG-3′ and 5′-GCAAATCCAAGTTTCTGAAGC-3′; 5′-GCACTTACTACTTACCATACC-3′, and 5′-ACCGCCAAGATACAA CTTGG-3′. The amplified products were purified with a GeneJet PCR purification kit (Thermo Fisher Scientific, Waltham, MA, USA) and sequenced at Macrogen Europe (Macrogen Europe BV).

### 2.5. Phylogenetic and Recombination Analysis

Using the MEGAx tool (10.2.6) [39], the sequences were translated into amino acids, and multiple sequence alignment was performed with the ClustalW plugin. The amino acid sequences, all with closest matches as well as from other genera within the viral family, were obtained from the NCBI protein database. The phylogenetic trees were constructed using a maximum likelihood model based on specific protein sequences using 500 bootstrap replicates. Similarity plots were generated with SimPlot [40], using a Kimura model, a window size of 600 bp, and a step size of 100 bp. The presence of recombination was examined by applying different models (RDP, GENECONV, Bootscan, MaxChi, Chimaera, SiScan, 3Seq, LARD, and Phylpro) using RDP5 (version 5.23) [41]. The recombination events were considered to be valid when the *p*-value was ≤0.05 for at least three different models and when the beginning and end breakpoints related to each recombination event could be determined.

### 2.6. Data Availability

The short paired-end sequence read data are available at the NCBI’s Sequence Read Archive (SRA) under BioProject number PRJNA823526, and the viral genomes are available at GenBank with accession numbers as represented in Supplementary Table S2.

## 3. Results

### 3.1. Overview of Bat Saliva- and Feces-Associated Viruses

Six saliva and nine fecal samples were collected from ten Pipisterllus pygmaeus bats trapped from roosts, from two different locations in Uppsala, Sweden (Table 1). Total RNA was extracted and processed for enriching viral nucleic acids prior to sequencing using the Illumina platform. Up to 90 million paired-end (PE) sequence reads were generated from each pool with most sequences obtained from fecal samples from the F-MV pool (Table 1). The taxonomic classification tool, Kaiju, classified 42–52% of the total reads in which bacteria and viruses represented 37–39% and 0.01–1.57% of the reads, respectively. A large number of reads were not classified. This was either due to the sequences having no significant matches or being too highly divergent to be able to classify using a similarity tool.

**Figure 1:**
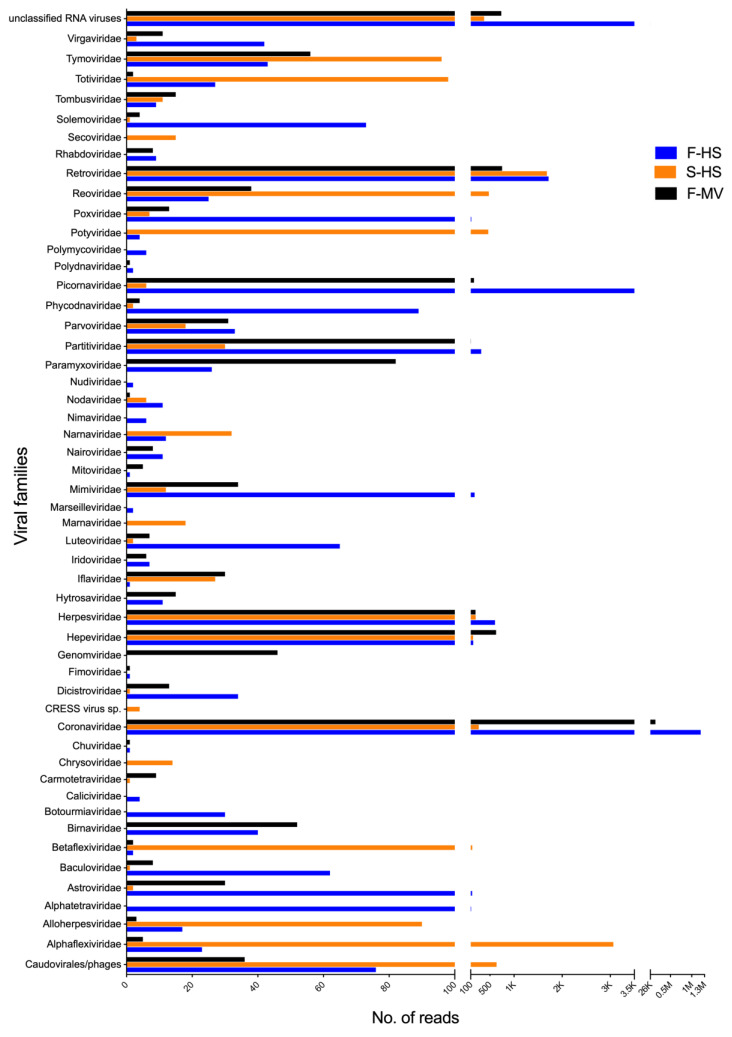
Schematic summary of the number of reads related to viruses classified by viral family at the protein level. Scale breaks were introduced in the x-axis for the visualization of the variable number of viral counts in each dataset. The exact number of viral counts are provided in Supplementary Table S1.

Figure 1 provides an overview of the viral sequences classified to different viral families. In total, sequences related to 51 viral families as well as to unclassified RNA viruses of bacterial, insect, fungal, mammalian, and plant infecting viruses were identified. The most frequently detected viruses, examining all pools, belonged to the families *Coronaviridae*, *Retroviridae*, *Herpesviridae*, *Hepeviridae*, *Tymoviridae*, and unclassified RNA viruses (Figure 1). Viral sequences from the families *Alphaflexiviridae*, *Betaflexiviridae*, *Potyviridae*, *Reoviridae*, and *Totiviridae* were more prevalent in saliva (S-HS), while *Alphatetraviridae*, *Astroviridae*, *Dicistroviridae*, *Luteoviridae*, *Iflaviridae*, *Mimiviridae*, *Paramyxoviridae*, *Partitiviridae*, *Picornaviridae*, *Poxviridae*, *Solemoviridae*, and *Virgaviridae*-related sequences were detected in feces (F-MV and F-HS pools). The exact viral read counts for each viral family are provided in Supplementary Table S1. The majority of the viral reads showed limited protein identity to previously known viruses in the database and therefore may represent the detection of novel viruses.

The assembly of good quality sequences generated several longer contigs (Supplementary Tables S3–S5), some of which represented near full-length and partial viral genomes, including coronaviruses, picornaviruses, and hepevirus. Therefore, selected viral genomes were further characterized and phylogentically analyzed. 

### 3.2. Bat Coronaviruses

**Figure 2:**
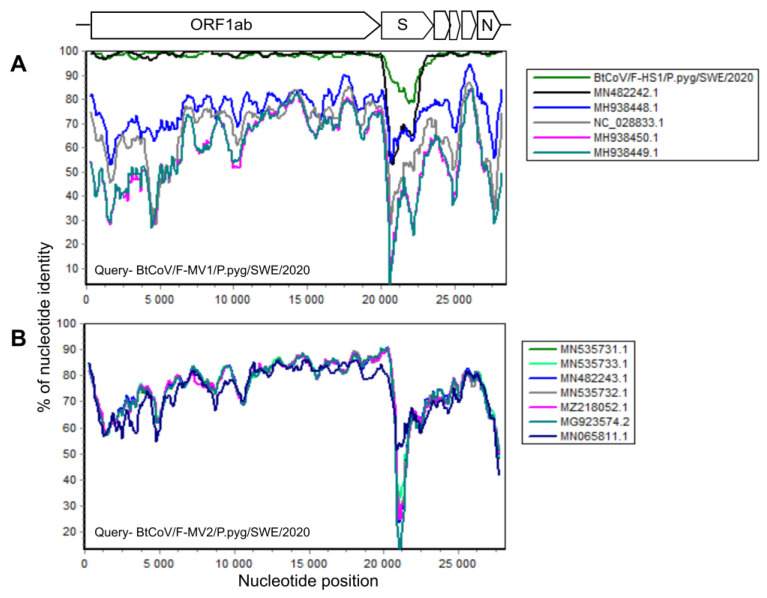
Simplot analysis of Swedish bat coronaviruses. Near full-length sequences of BtCoV/F-MV1/P.pyg/SWE/2020 (**A**) and BtCoV/F-MV2/P.pyg/SWE/2020 (**B**) were used as queries and compared with closest reference sequences in the SimPlot similarity analysis. All the analyses were performed with a Kimura model, a window size of 600 base pairs, and a step size of 100 base pairs.

**Figure 3:**
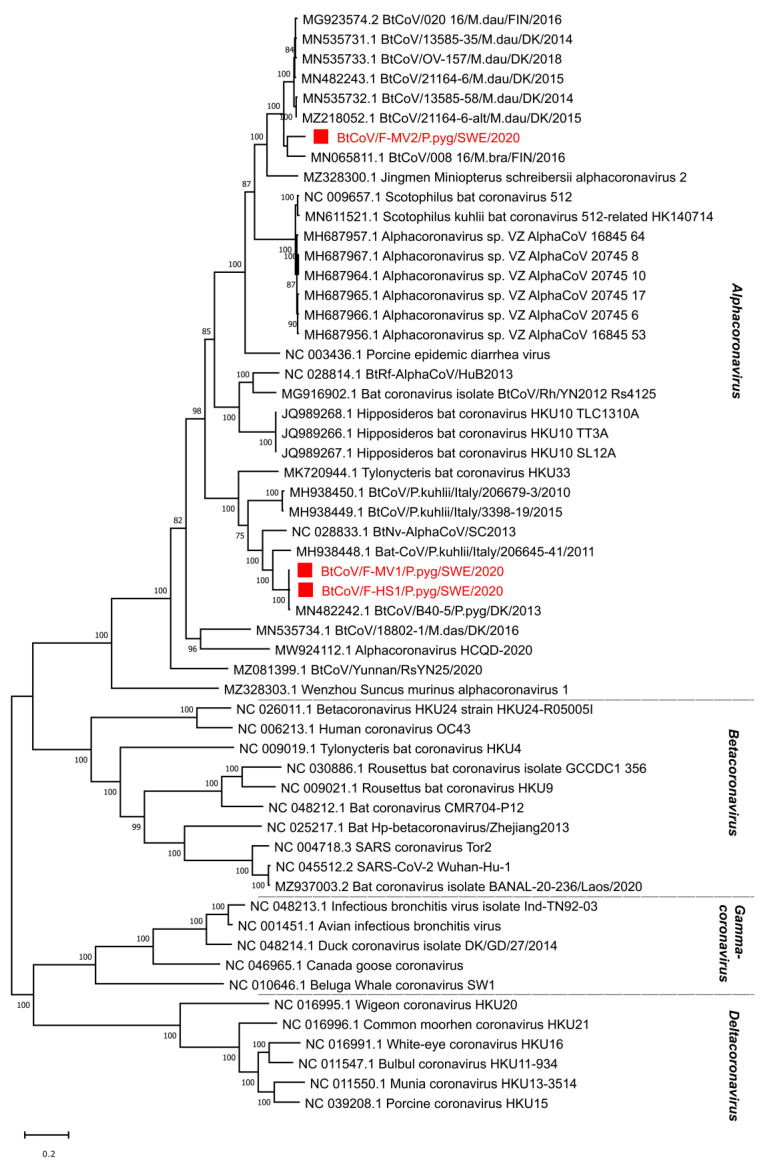
Phylogenetic analysis of coronaviruses from Sweden based on predicted full-length *ORF1ab* protein. The sequences reported in this study, BtCoV/F-MV1/P.pyg/SWE/2020 (ON457560), BtCoV/F-MV2/P.pyg/SWE/2020 (ON457561), and BtCoV/F-HS1/P.pyg/SWE/2020 (ON457562), (indicated by red square boxes and red text), and the closest sequence matches from Genbank and ICTV reference sequences from each genus within the *Orthocoronavirinae* subfamily are included in the analysis. The tree was generated using a maximum likelihood model with 500 bootstrap iterations on ClustalW-aligned amino acid sequences, with a total of 8037 amino acid positions in the final dataset. Only bootstrap values above 50 are displayed on the nodes.

In total, there were 145,383 corona viral reads in the F-MV pool and 226 reads in the S-HS pool. Most coronavirus reads, 1,210,402, were found in the F-HS pool, accounting for 1.51% of the total reads. From the F-MV pool, two near full-length coronavirus genomes were obtained through Megahit *de novo* assembly and Sanger sequencing. The near full-length bat coronavirus (BtCoV) genome (BtCoV/F-MV1/P.pyg/SWE/2020, GenBank accession number ON457560) consisted of 27,865 nucleotides (nt), encoding for all the coronavirus reference proteins (ORF1ab, S, ORF3, E, M, N), partial 5′ UTR (231 nt), and 3′ UTR (674 nt). The BLAST search of individual ORFs showed that this genome was most similar to the Danish BtCoV (MN482242.1) from *P. pygmaeus* bats [42], with an identity of 89–99% at protein level. The second near full-length BtCoV genome (BtCoV/F-MV2/P.pyg/SWE/2020, GenBank accession number ON457561) was generated from four contigs ranging from 558 to 13,376 nt, covering 98% of the reference genome with 50–300 nt gaps in the ORF1ab. The gaps were filled with Sanger sequencing using specific PCR primers designed from the contigs, and this generated a longer contig consisting of 27,944 nt, encoding all the coronaviral reference proteins, partial 5′ (321 nt), and 3′ (372 nt) UTR. The individual ORFs of this genome shares 78–99% identity at protein level with other BtCoVs from Denmark (MN535733.1 and MZ218060.1) [42] and Finland (MG923573.2 and MN065811.1). Interestingly, both the coronaviruses from this pool (F-MV) are divergent from each other and share 65–73% identity at nucleotide level. From the F-HS pool, a near full-length genome (BtCoV/F-HS1/P.pyg/SWE/2020, Genbank accession number ON457562) was assembled, and this genome consisted of 27,943 nt and contained all the expected reference viral ORFs as well as partial 5′ (250 nt) and 3′ (731 nt) UTR ends. The individual ORFs of this genome, using BLAST search, showed closest protein identity (88–100%) to Danish BtCoV (MN482242.1) [42] with the highest variation in spike protein. The sequence similarity of the identified BtCoVs to other alphacoronaviruses was examined by SimPlot analysis (Figure 2A,B) and pairwise alignment. The genomic organization of the characterized BtCoVs in this study was similar to that of other alpha BtCoVs. BtCoV/F-MV1/P.pyg/SWE/2020 and BtCoV/F-HS1/P.pyg/SWE/2020 were most closely related to the Danish BtCoV (MN482242.1) (Figure 2A). The BtCoV/F-MV2/P.pyg/SWE/2020 had closest identity to BtCoVs from Finland and Denmark (Figure 2B). The occurrence of recombination was tested with RDP5, using the complete genomes of alpha BtCoVs. This analysis suggests the absence of recombination for BtCoV/F-MV1/P.pyg/SWE/2020 and BtCoV/F-HS1/P.pyg/SWE/2020. However, a recombination event was observed in ORF1a of BtCoV/F-MV2/P.pyg/SWE/2020 at nucleotide positions 7220–7874. This event was detected by six different methods (*p*-values ≤0.05); the *p*-values from each method are shown in Supplementary Table S6. The major and minor parents for this recombination event were Danish (MN535732.1) and Finnish (MG923574.2) BtCoVs, respectively. To further characterize the Swedish BtCoVs, the full ORF1ab protein coding sequences were translated in silico, and the amino acid sequences were aligned with other coronaviruses from the *Alphacoronavirus*, *Betacoronavirus*, *Gammacoronavirus*, and *Deltacoronavirus* genera. The phylogenetic analysis of full-length ORF1ab protein showed that Swedish BtCoVs sequences from this study clustered with viruses within the Alphacoronavirus genus. Two of the BtCoVs, BtCoV/F-MV1/P.pyg/SWE/2020 and BtCoV/F-HS1/P.pyg/SWE/2020, were closely clustered with other BtCoVs from Denmark and Italy within the subgenus *Nyctacovirus*, and BtCoV/F-MV2/P.pyg/SWE/2020 grouped with Finish and Danish BtCoVs within the subgenus *Pedacovirus* BtCoVs (Figure 3). From saliva (S-HS), 266 reads were classified in the *Coronaviridae* viral family, which produced nine shorter contigs ranging from 314 to 1059 nt; these contigs shared 89–99% protein identity to Danish BtCoV (MN482242.1).

### 3.3. Bat Picornaviruses

**Figure 4:**
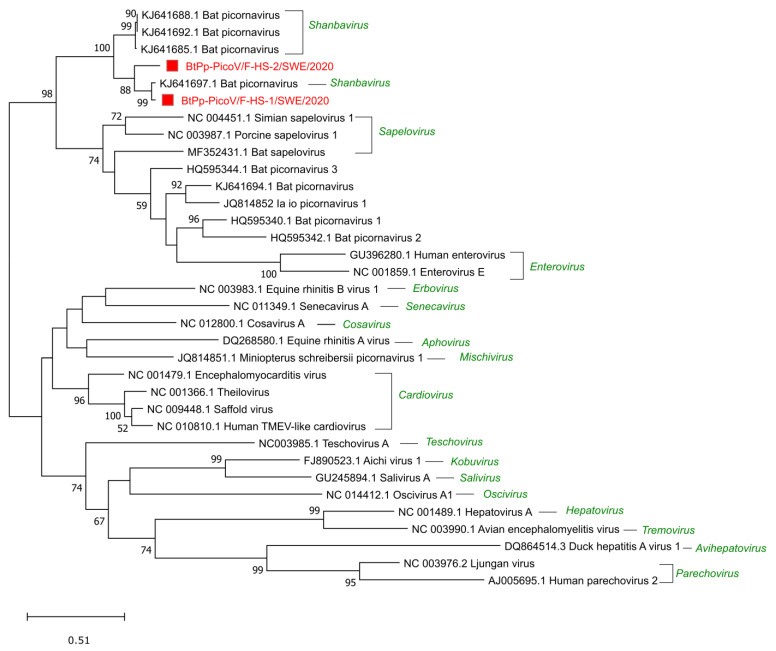
Phylogenetic analysis of bat picornaviruses from Sweden based on the conserved capsid protein. The sequences reported in this study, BtPp-PicoV/F-HS1/SWE/2020 (ON375332) and BtPp-PicoV/F-HS2/SWE/2020 (ON375333) (indicated by red square boxes and red text), the closest sequence matches from Genbank and ICTV reference sequences from a different genus within the *Picornaviridae* family (indicated in green) are included in the analysis. The tree was generated using MEGAx and a maximum likelihood model with 500 bootstrap iterations on ClustalW aligned amino acid sequences, with a total length of 256 amino acids. Only bootstrap values above 50 are displayed on the nodes.

Sequences related to the *Picornaviridae* family were detected in all the samples, 6 reads in S-HS, 170 reads in the F-MV pool, and the most in the F-HS pool with 23,858 reads. A near full-length genome and a partial bat picorna viral genome (BtPVs) were assembled from the F-HS pool and further analyzed for phylogenetic relationships. The near full-length BtPV genome (BtPp-PicoV/F-HS-1/SWE/2020, GenBank accession number ON375332) consists of 7033 nt and encodes a long single polyprotein (2207 amino acids) that is flanked by partial UTRs (5′ (336 nt) and 3′ (76 nt)). This genome shared the closest identity of 83% and 94% at the nucleotide and protein levels, respectively, to a BtPV (KJ641697.1) identified in *Nyctalus valutinus* bats from China [7]. The partial BtPV (BtPp-PicoV/F-HS-2/SWE/2020, 5752 nt, GenBank accession number ON375333) consisted of partial 5′ UTR and a near full-length polyprotein (1924 amino acids), which shared 78% and 88% identity at the nucleotide and protein level with BtPV (KJ641697.1). The two BtPp-PicoV/F-HS-1/SWE/2020 and BtPp-PicoV/F-HS-2/SWE/2020 show 82% nucleotide identity between each other, with nucleotide mismatches throughout the genomes, indicating two different strains of BtPVs in F-HS. The phylogenetic analysis using the highly conserved capsid protein shows that these genomes grouped with other BtPVs within the *Shanbavirus* genus in the *Picornaviridae* family and closely with BtPV (KJ641697.1) from China (Figure 4). Seven contigs ranging from 302 to 1506 nt were related to bat picornaviruses from the F-MV pool, and all the contigs shared protein identity (91–98%) to the same BtPV as shown with the genomes from the F-HS pool.

### 3.4. Bat Hepeviruses

**Figure 5:**
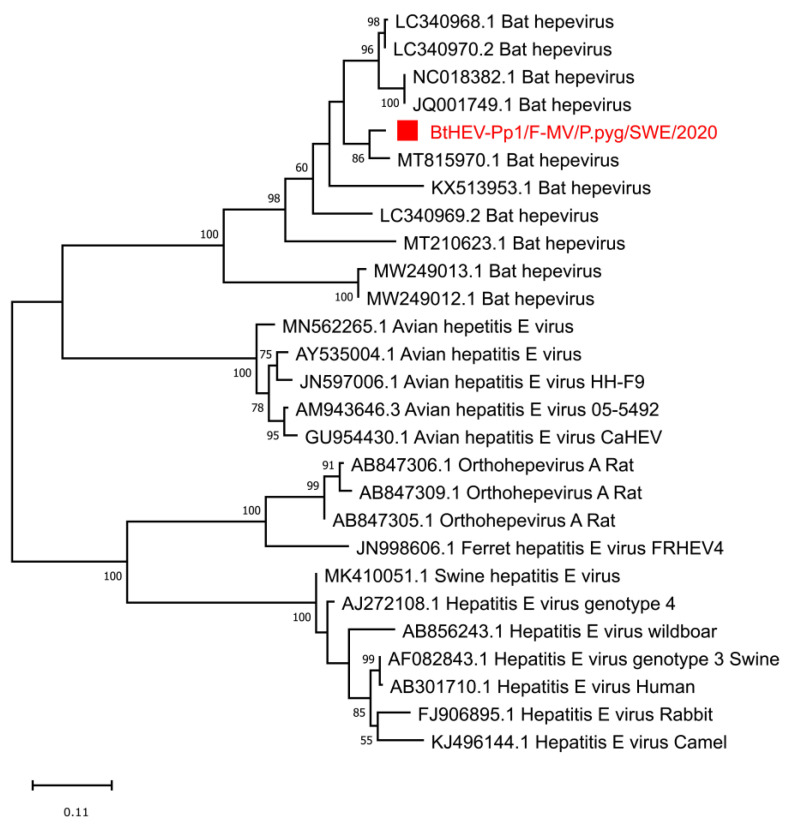
Phylogenetic analysis of bat hepevirus from Sweden based on predicted conserved RdRp protein. The sequence reported in this study, BtHEV-Pp1/F-MV/P.Pyg/SWE/2020 (ON513427) (indicated by red square boxes and red text), and the closest sequence matches from Genbank and ICTV reference sequences from within the *Hepeviridae* family are included in the analysis. The tree was generated using MEGAx and a maximum likelihood model with 500 bootstrap iterations on ClustalW aligned amino acid sequences, with a total length of 351 amino acids. Only bootstrap values above 50 are displayed on the nodes.

Viral reads classified as the *Hepeviridae* family were identified in all the pools, ranging from 148 to 627 reads. From the F-MV pool, four longer contigs were generated (BtHEV-Pp1/F-MV/P.pyg/SWE/2020, Genbank number ON513427), and these contigs showed the closest protein identity (82–96%) to the non-structural and capsid proteins of a known bat hepevirus (MT815970.1) previously identified in *P. nathusii* bats from Switzerland and covering approximately 85% of ORF1 in the reference genome, including the RNA-dependent RNA polymerase (RdRp) domain. No contigs related to hepeviruses could be assembled from S-HS and the F-HS pool. The multiple sequence alignment and phylogenetic analysis using the conserved RdRp protein from other hepeviruses in the family showed that the identified sequence clustered with bat hepeviruses and closely with *P. nathusii* bat hepevirus (Figure 5).

### 3.5. Other Viruses

Altogether, 1,421,206 reads were taxonomically classified to 51 viral families and to unclassified RNA viruses, as shown in Supplementary Table S1. The assembled contigs of these viral reads represent partial viral genomes that show similarities with viruses from bats, insects, and plants. The list of contigs and their identities are shown in Supplementary Tables S2–S4. The majority of the contigs show low identity to unclassified RNA viruses. Many of these unclassified viruses are viruses identified in insects from China through different metagenomics studies [43,44]. Reoviral contigs were assembled from all the pools; in the F-MV pool, 13 contigs were assembled showing protein identity of 24–65% to structural and nonstructural proteins of known reoviral segments characterized from different insects. The sizes of the contigs are in the range between 427 and 4106 nt and may represent the full or near full-length segments of a novel reovirus, which needs to be further determined. Three alphatetraviral contigs were assembled showing protein identity (42–59%) to the RdRp region of the Helicoverpa armigera stunt virus (KX423453.1). A longer contig of 6286 nt shared the closest identity to a polyprotein of the unclassified dicistrovirus, Praha dicistro-like virus 2, with an identity of 32% at the protein level. Another longer contig of 12,959 nt, classified as an unclassified RNA virus, showed low similarity (24% identity at the protein level) to the Botrytis cinerea negative-stranded RNA virus 1 (NC_028466.3). In both the F-MV and F-HS pools, nine astrovirus-related contigs ranging from 332 to 1138 nt were found, and these showed protein similarity (42–91%) with other bat astroviruses. In total, six parvovirus contigs (361–3367 nt in size) were obtained. These contigs had a protein similarity of 41–87% to different bat adeno-associated viruses. In the F-MV pool, eight assembled contigs with lengths between 325 and 826 nt showed protein identity (35–87%) to the polymerase region of a bat paramyxovirus (KJ641657.1). In addition, contigs related to viral families including *Tymoviridae, Partitiviridae, Nodaviridae, Totiviridae, Carmotetraviridae, Alphaflexiviridae,* and *Microviridae* were obtained, as were those that matched different unclassified RNA viruses.

## 4. Discussion

Characterizing bat virome is of great importance to increase our knowledge on viral diversity, evolution, viral host range, as well as the transmission of bat viruses among the species. This is the first study to analyze the *P. pygmaeus* bat virome in Sweden. Saliva and fecal samples from *P. pygmaeus* bats were enriched for viruses and analyzed using viral metagenomics, a target-free and unbiased method. Despite some limitations [45], this method is able to detect all viruses present in a sample with no prior information of viruses. Metagenomic characterization of bat viruses from fecal samples represents an ideal non-invasive method for monitoring viral diversity at the colony level and discovering novel viruses with zoonotic potential. Similar metagenomic studies of bats have discovered new bat viruses and analyzed viral diversity in different countries [27,28,29]. The results from this study show that a wide variety of viruses, belonging to 51 viral families and unclassified RNA viruses, were present in *P. pygmaeus* bats from Uppsala, Sweden. The identified viruses include eukaryotic, insect, and plant viruses. For some of these viruses, the near-complete genomes were genetically characterized, while for others, only the partial genomes and individual reads were obtained. More viral reads were obtained from fecal samples as compared with the saliva samples, with differences in both diversity and composition. These observations are consistent with other studies [28]. The majority of the viral reads in the two fecal pools belonged to eukaryotic viruses related to viruses identified in different insects and plants as observed in other bat virome studies [19,27,28], possibly representing the dietary habit of the *P. pygmaeus* bats (insectivorous) and further highlighting the bat’s bio-insecticidal role in the environment. In saliva, many viral reads related to viruses identified in fungi and plants were obtained, such as *Alphaflexiviridae*, *Potyviridae*, *Totiviridae,* and *Tymoviridae*, which might reflect the plant-based diet eaten by insects. Moreover, viruses from the *Astroviridae*, *Herpesviridae*, *Paramyxoviridae*, *Poxviridae,* and *Retroviridae* families, which all have members with zoonotic potential, were identified in this study (Supplementary Table S1). However, the host-range of the identified viruses is yet to be determined. Although poxviruses have been identified previously in bats [46,47], these sequences may represent different viral enzymes or show high similarity to their respective mammalian analogs as they interact with the host immune system. Retroviral sequences have been shown to be associated with their mammalian hosts for a long evolutionary period and have been integrated into the host genomes in the form of endogenous retroviruses [48,49], and therefore, the detected virus reads could, for example, represent contaminating host DNA; however, this needs to be evaluated.

Bats have been recognized as natural reservoirs for coronaviruses in the genera *Alphacoronavirus* and *Betacoronavirus*, which also include a broad range of mammalian infecting coronaviruses, for example, PEDV, MERS-related, SARS, and SARS-CoV-2. Previous studies from Sweden, Denmark, and Finland have reported bat coronaviruses in *Myotis daubentonii*, *Myotis dasycneme*, *Myotis brandtii,* and *Pipistrellus pygmaeus* [26,32,42]. However, the underreporting of coronavirus sequences in northern Europe is most likely due to limited sampling. In this study, three near full-length genomes (BtCoV/F-MV1/P.pyg/SWE/2020, BtCoV/F-HS1/P.pyg/SWE/2020, and BtCoV/F-MV2/P.pyg/SWE/2020) of coronaviruses were characterized from *P. pygmaeus* bats from two different locations in Uppsala, Sweden. The genome organization of the characterized genomes is the same as those for alphacoronaviruses [50]. The Swedish coronavirus sequences were highly similar (up to 99%) to Danish coronaviruses detected in *P. pygameus* and *M. daubentonii* bats [42]. The recombination events in these genome sequences were analyzed using RDP5, and a single recombination event was detected in the BtCoV/F-MV2/P.pyg/SWE/2020 genome. BtCoV/F-MV1/P.pyg/SWE/2020 and BtCoV/F-HS1/P.pyg/SWE/2020 were phylogenetically related to BtCoV viruses from Denmark and Italy, and to the ICTV reference genomes in the *Nyctacovirus* subgenus, *Pipistrellus kuhlii* coronavirus 3398 and *Nyctalus velutinus* alphacoronavirus SC-2013 [51]. BtCoV/F-MV2/P.pyg/SWE/2020 was closely related to BtCoV from *M. daubentonii* from Finland and Denmark and to the ICTV reference genomes in the *Pedacovirus* subgenus, PEDV and *Scotophilus* BtCoV 512 [51]. These results suggest that the identified sequences belong to the *Nyctacovirus* and *Pedacovirus* subgenera within the genus *Alphacoronavirus*. The distribution and diversity of coronaviruses in bats are high. This may be explained by genetic mutations and recombination, as well as the host competence for infections and co-infections [7,52,53]. More than 60 coronavirus species have been reported in 14 of 21 bat families across all continents except Antarctica [17], suggesting a long co-evolutionary relationship.

Picornaviruses of the family *Picornaviridae* are small, non-enveloped, positive sense, single-stranded RNA viruses with a genome size of 7–9 kb [54]. The viruses in this family have a broad range of hosts, including vertebrates, insects, and plants. These viruses are diverse and able to transmit by several routes and can cause diseases such as encephalitis, hepatic, gastroenteric, neurological, and respiratory diseases in different vertebrate hosts [55,56]. A near full-length (BtPV/F-HS-1/P.pyg/SWE/2020) and a partial genome (BtPV/F-HS-2/P.pyg/SWE/2020) of picornaviruses were characterized from the F-HS pool, which showed close similarity of 94% and 88% to a bat picornavirus (KJ641697) from the Chinese *Nyctalus velutinus* bats and grouped phylogenetically with the other bat picornaviruses from the *Shanbavirus* genus. The near full-length picornavirus had the same genome organization and shared the genetic features described in the ICTV guidelines for the *Picornaviridae* family [51]. In the F-MV pool, shorter contigs were obtained which showed closest identity (91–98% at the aa level) to the same picornavirus as in the F-HS pool. The present analysis shows that a large number of bat-associated picornaviruses exist in Swedish bats, as has been previously reported from Asia, Europe, Africa, and the USA [7,18,27,57], suggesting that these viruses are broadly distributed across the globe. It has been shown that a particular picornavirus or highly similar strains can infect bats from different genera or species [58]. This also suggests that bat picornaviruses could cross the species or genus barrier among the bats and may become permissible for new hosts. It remains to be determined whether the existence of the shanbaviruses follows a particular geographical pattern and can be linked to widespread *Pipistrellus* and associated bat species.

Hepeviruses are small, non-enveloped, positive sense, single stranded RNA viruses with a genome size from 6.6 to 7.2 kb. These viruses have, so far, been identified only in vertebrate hosts such as mammals (*Orthohepevirus A, C,* and *D*), birds (*Orthohepevirus B*), and trout (*Piscihepevirus*) [59]. Viruses in this family can cause severe diseases in the host. For example, hepatitis E virus (HEV) in humans and several mammalian species and avian HEV in chickens can cause acute hepatitis. Four contigs from the F-MV pool in this study, covering 85% of a hepevirus genome, showed the highest identity to the ORF1 region of a bat hepevirus identified in Switzerland [29]. This genome phylogenetically clustered with other unclassified bat hepeviruses and *Orthohepevirus D* viruses. Bats have previously been shown to harbor different hepeviruses, and they are distributed geographically in different species [29,60,61,62].

In addition, a large number of viral reads and contigs was not assigned to any known viral family (unclassified), and many showed low identity to known viruses. These may represent novel (previously uncharacterized) viruses, and further studies are needed to both genetically and biologically characterize these viruses.

## 5. Conclusions

The viral metagenomic data presented in this study provide an overview of the virome of the Swedish *P. pygmaeus* bat population from two locations in Uppsala, Sweden. Sequences related to 51 viral families and unclassified viruses were identified, and genomes of alphacoronaviruses and picornaviruses were characterized. This study significantly contributes to a better understanding of the global diversity of bat viruses in general. Further studies are recommended to characterize the bat virome from different sample types, bat species, and locations, which will increase our knowledge of the viral diversity, transmission, and evolution of bat viruses, as well as help predict future emerging viruses circulating in Sweden.

## Figures and Tables

**Table 1:** Summary of *Pipistrellus pygmaeus* bat samples analyzed in this study, taxonomic classification of reads and assembly of the reads.

	F-MV	S-HS	F-HS
Collection date	11-Jun-20	09-Jul-20	09-Jul-20
Sample type	Feces	Saliva	Feces
No. of bats included	4	6	5
Sequence output	90,906,601	79,365,429	80,303,677
Good quality PE reads	89,601,219	78,515,127	78,967,253
Good quality PE reads %	98.5	98.9	98.3
Classified (% of raw data)	38,498,376 (42.34%)	46,208,544 (58.22%)	41,447,487 (51.61%)
Eukaryotes	2,090,656 (2.3%)	727,840 (0.92%)	9,764,846 (12.16)
Bacteria	36,257,800 (39.88%)	45,469,019 (57.29%)	30,416,093 (37.88%)
Archea	1062 (0.001%)	3129 (0.004%)	1251 (0.002%)
Viruses	1,488,551 (0.16%)	7,956 (0.01%)	1,264,699 (1.57%)
Assembled contigs	48,618	12,038	43,029
Average contig length	662	792	662
Classified contigs	25,670	8459	20,753
Viral contigs	102	105	82

F-MV: feces from Hammarskog location 1; S-HS and F-HS: saliva and feces, respectively, from Hammarskog location 2.

## Data Availability

The short paired-end sequence read data are available at the NCBI’s Sequence Read Archive (SRA; https://www.ncbi.nlm.nih.gov/sra) under BioProject number PRJNA823526, and the viral genomes are available at GenBank (https://www.ncbi.nlm.nih.gov/nucleotide/) with accession numbers as represented in Supplementary Table S2.

## Supplementary Materials

The following supporting information can be downloaded at: https://www.mdpi.com/article/10.3390/v14081654/s1, Table S1: Viral read counts belonged to each family from F-MV, S-HS and F-HS pools. Table S2: the viral genomes characterized in this study with GenBank with accession numbers and genome lengths. Table S3: Contigs classified as viruses from F-MV. Table S4: Contigs classified as viruses from S-HS. Table S5: Contigs classified as viruses from F-HS. Table S6: Recombination event in BtCoV/F-MV2/P.pyg/SWE/2020, detected by RDP5.
